# Research and application of intelligent image processing technology in the auxiliary diagnosis of aortic coarctation

**DOI:** 10.3389/fped.2023.1131273

**Published:** 2023-02-23

**Authors:** Taocui Yan, Jinjie Qin, Yulin Zhang, Qiuni Li, Baoru Han, Xin Jin

**Affiliations:** ^1^ Medical Data Science Academy, College of Medical Informatics, Chongqing Medical University, Chongqing, China; ^2^ Department of Radiology, Children's Hospital of Chongqing Medical University, Chongqing, China; ^3^ Technology Research and Development Department of Chongqing Intech Technology Co., LTD, Chongqing,, China; ^4^ Department of Cardiothoracic Surgery, Ministry of Education Key Laboratory of Child Development and Disorders, China International Science and Technology Cooperation Base of Child Development and Critical Disorders, National Clinical Research Center for Child Health and Disorders, Chongqing Key Laboratory of Pediatrics, Children's Hospital of Chongqing Medical University, Chongqing, China

**Keywords:** coarctation of the aorta (COA), computed tomography angiography (CTA), intelligent image processing, intelligent measurement, auxiliary diagnosis

## Abstract

**Objective:**

To explore the application of the proposed intelligent image processing method in the diagnosis of aortic coarctation computed tomography angiography (CTA) and to clarify its value in the diagnosis of aortic coarctation based on the diagnosis results.

**Methods:**

Fifty-three children with coarctation of the aorta (CoA) and forty children without CoA were selected to constitute the study population. CTA was performed on all subjects. The minimum diameters of the ascending aorta, proximal arch, distal arch, isthmus, and descending aorta were measured using manual and intelligent methods, respectively. The Wilcoxon signed-rank test was used to analyze the differences between the two measurements. The surgical diagnosis results were used as the gold standard, and the diagnostic results obtained by the two measurement methods were compared with the gold standard to quantitatively evaluate the diagnostic results of CoA by the two measurement methods. The Kappa test was used to analyze the consistency of intelligence diagnosis results with the gold standard.

**Results:**

Whether people have CoA or not, there was a significant difference (*p* < 0.05) in the measurements of the minimum diameter at most sites using the two methods. However, close final diagnoses were made using the intelligent method and the manual. Meanwhile, the intelligent measurement method obtained higher accuracy, specificity, and AUC (area under the curve) compared to manual measurement in diagnosing CoA based on Karl's classification (accuracy = 0.95, specificity = 0.9, and AUC = 0.94). Furthermore, the diagnostic results of the intelligence method applied to the three criteria agreed well with the gold standard (all kappa ≥ 0.8). The results of the comparative analysis showed that Karl's classification had the best diagnostic effect on CoA.

**Conclusion:**

The proposed intelligent method based on image processing can be successfully applied to assist in the diagnosis of CoA.

## 1. Introduction

Coarctation of the aorta (CoA) is one of the most challenging and crucial congenital heart diseases to diagnose (1), with an incidence of approximately 4 per 10,000 live births (2). It can lead to more severe cardiovascular complications and even death (3). However, if successfully diagnosed early, it can be repaired promptly through surgery or percutaneous balloon angioplasty and stenting. Therefore, diagnosis and intervention of patients in a timely, as well as regular postoperative follow-up, are an indispensable part of reducing the risk of aortic coarctation and improving its cure rate.

81% of patients diagnosed with aortic coarctation are often complicated by aortic arch hypoplasia (HAA) (4), with more complex pathological changes and clinical symptoms, resulting in inconsistency in their diagnostic and therapeutic options (5, 6).The 2020 Expert Consensus on Surgical Treatment of Congenital Heart Disease in China specifies four diagnostic criteria for aortic arch hypoplasia (HAA) based on morphological analysis: congenital heart disease database classification (CHD database) (7), Karl's classification (8), Langley's classification (9), and Brouwer's classification (10). Based on the above diagnostic criteria, the diagnosis of aortic coarctation not only requires an experienced imaging physician but is also highly subjective, which profoundly affects the accuracy of disease diagnosis, so there is a great need for more intelligent methods to achieve a rapid diagnosis of aortic coarctation.

Recently, three-dimensional (3D) models have brought increasing advantages for the diagnosis of CoA (11), and several studies have been devoted to the automatic 3D segmentation of the aorta to make rapid diagnostic decisions through more intuitive morphological analysis (11–13). On this basis, if measurements of diameter at any part of the aortic 3D model could be obtained automatically could improve the accuracy of imaging analysis and reduce diagnostic subjectivity (14). However, few studies have been performed to automatically construct and measure the aortic diameter. Although Gamechi et al. (12) measured the diameter of the ascending aorta and descending aorta based on non-enhanced CT after successful automated segmentation of the aorta, the method has not been validated in people with aortic disease.

The abovementioned four CoA diagnostic criteria require measurements of aortic diameter, so this study proposes a method based on intelligent image processing techniques for measuring the minimum diameter of the aorta. We compare the diameter sizes and diagnostic results of manual and intelligent measurements using surgical diagnosis as the “gold standard”, and the accuracy of the three diagnostic criteria was subsequently analyzed to assist imaging physicians and clinicians in the accurate diagnosis and efficient treatment of aortic coarctation.

## 2. Materials and methods

### 2.1. Overview of diagnostic criteria

CoA is classified by the Congenital Heart Surgery Nomenclature and Database Project (15) into three categories: isolated coarctation, coarctation and ventricular septal defect (VSD), and coarctation and complex intracardiac anomaly. According to the range and degree of coarctation, CoA can be divided into simple CoA and aortic arch dysplasia. The methods for judging its diagnostic criteria are summarized as follows:

(1) CHD database classification: The diameters of the proximal arch, distal arch, and isthmus of the aorta are less than 60%, 50%, and 40% of the diameter of the ascending aorta, respectively (16).
(2) Karl's classification: Transverse arch diameter (mm) < weight (kg) + 1, mainly for newborns or small infants (8).
(3) Langley's classification: The diameter of the transverse arch is less than 50% of the diameter of the descending aorta (9).
(4) Brouwer's classification: The Z value of the diameter of the proximal aortic arch is less than −2 (10).

As there is no standard for Z-value in China, this paper uses the remaining three criteria to diagnose aortic coarctation. For Karl's and Langley's classification, it is necessary to note that the diameter of the narrowest part of the aortic arch is used for calculation.

### 2.2. Study population

**Figure 1:**
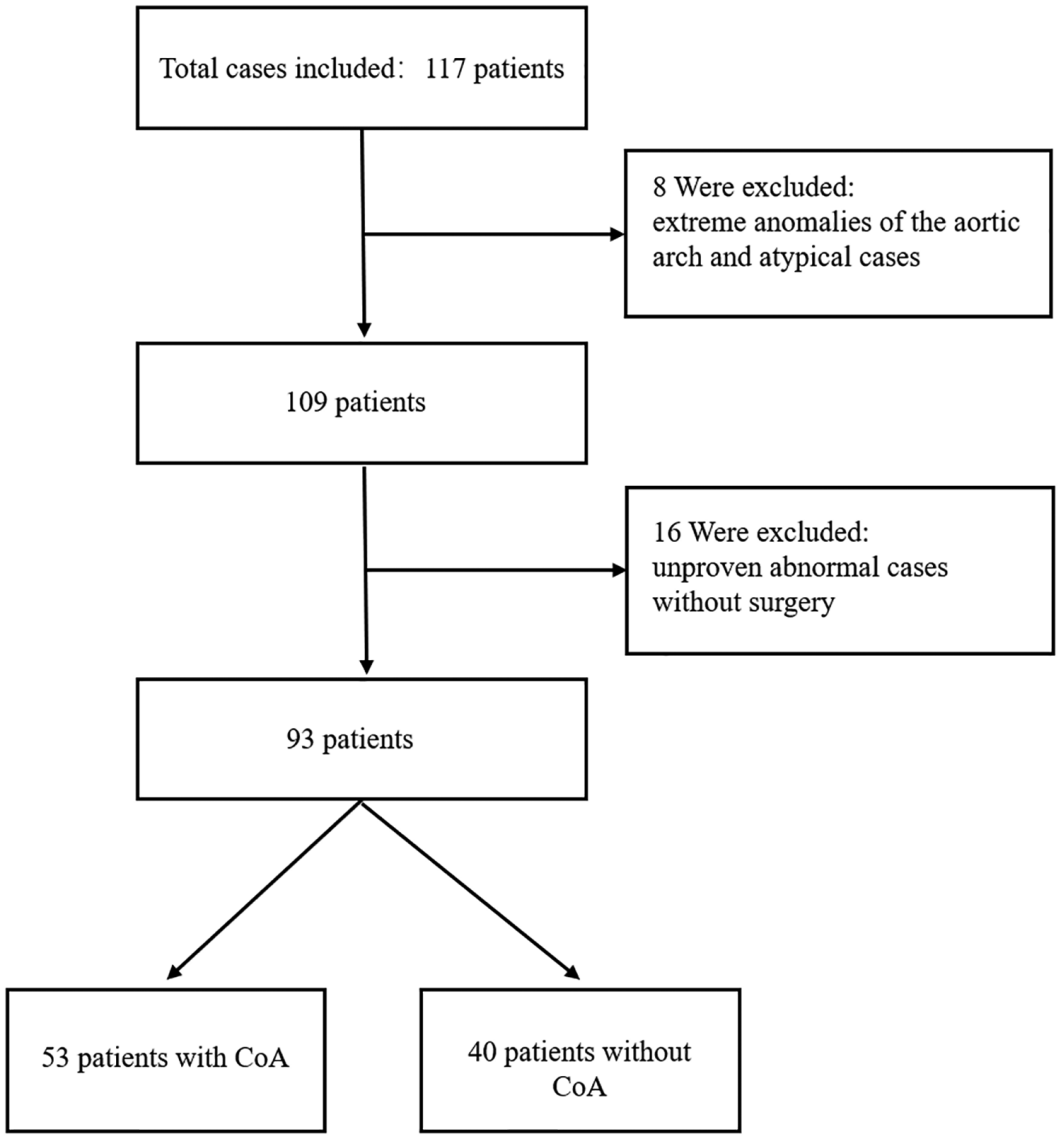
The flow chart of study population selection.

Data were collected from seventy-one children with CoA and forty-six children without CoA who attended the Children's Hospital of Chongqing Medical University between June 2018 and December 2020 in this study. All children with CoA were under one year of age, and their ages obeyed a normal distribution. Among them, two patients with extreme anomalies of the aorta and sixteen patients with unproven CoA without surgery were excluded from this research. In addition, six atypical patients without CoA were excluded. Finally, fifty-three children with CoA and forty without CoA constituted the study population (Figure 1).

### 2.3. Computed tomography angiography and manual measurement

To improve the early diagnosis of CoA, several diagnostic tests have been used in clinical practice. Currently, cardiac ultrasound is a routine test for CoA, and studies to improve the prenatal diagnosis of CoA have recently been conducted based on it (17, 18). However, since the aortic coarctation occurs mainly in the isthmus and its physical changes are not clear, it is often examined with the help of CT and MRI. MRI is also widely used to assess CoA (19), but due to its time-consuming, costly, and low spatial resolution, it has limitations compared with CT (20, 21). Therefore, in this study, CTA was used for all study subjects, and initial reconstruction of the scanned images was completed using image post-processing techniques.

Children who were hemodynamically unstable and uncooperative were sedated before CTA by oral 10% chloral hydrate (0.5 ml/kg body mass) or intramuscular sodium phenobarbital injection (5 ml/kg body mass), with careful monitoring of heart rate and saturation by the anesthesia team during sedation. A Philips Brilliance ICT machine was used to perform CT scanning from the lower neck to the level of the diaphragm, and the scanning parameters were set according to the ALARA principle: tube voltage 80–100 kV, tube current 35–85 mAs, pitch 0.2 mm, layer spacing 5.0 mm, layer thickness 5.0 mm, and image reconstruction layer thickness 1.0 mm. Iohexol 300 (mgI/ml) and iodixanol (270 mgI/ml) were injected into the dorsal vein of the hand and foot using a high-pressure syringe at a dose of 2 ml/kg and an injection rate of 0.6–3.0 ml/s. Phase II enhancement scans were performed 15–30 s and 50–60 s after drug administration, respectively.

The minimum internal diameters of the ascending aorta (AOA), proximal arch (D1), distal arch (D2), isthmus (D3), and descending aorta (DA) were measured using a double-blind method by two physicians who have been involved in cardiovascular disease research for many years, and each measurement was taken twice and averaged.

### 2.4. Acquisition of image data

**Figure 2:**
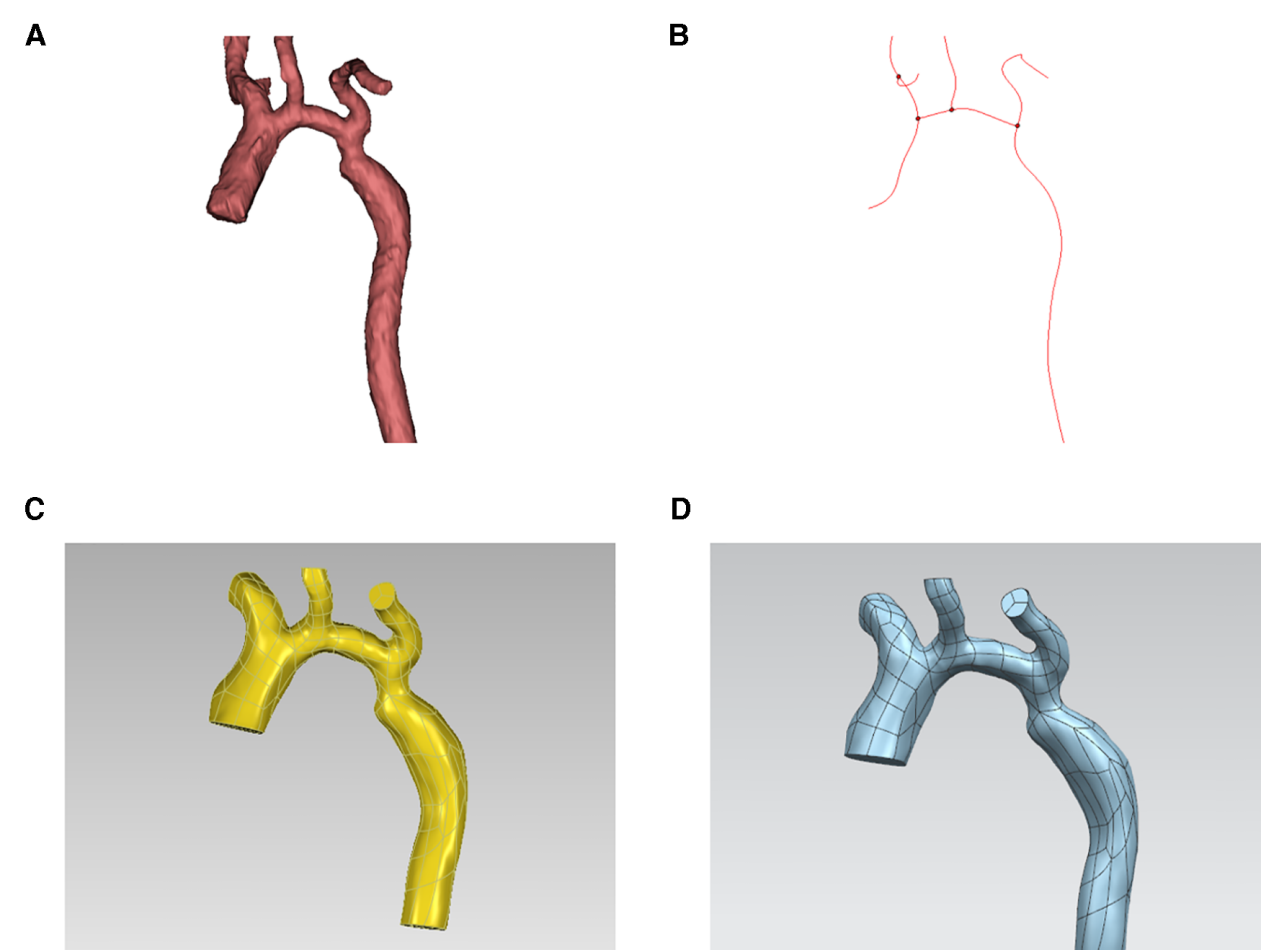
Acquisition of image data. (**A**) 3D (three-dimensional) aortic image after accurate reconstruction with mimics software. (**B**) Center line. (**C**) 3D aortic images after smoothing and surfacing process. (**D**) Final model.

**Figure 3:**
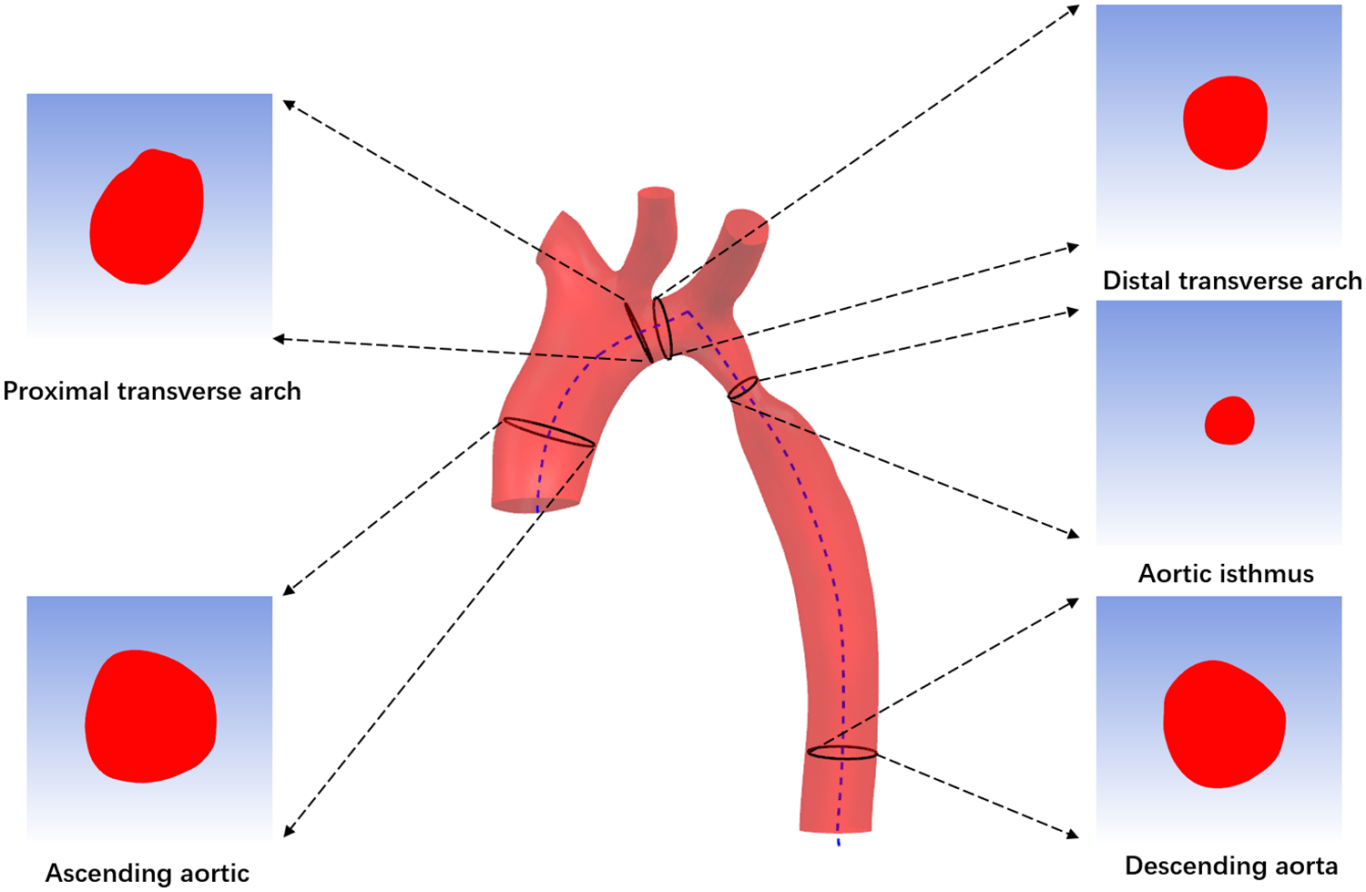
Acquisition of image data.

First, import CTA image data into Mimics 19.0 Image Workstation in DICOM format, select the “Segment” function module and use the “CT Heart” command under “Cardiovascular” to set the threshold range of 283Hu-2750Hu for threshold segmentation. Click “Calculate” to obtain the segmented image and select the aortic region to construct a rough stereoscopic model of the aorta. After that, use the “lasso” command in “Edit Masks” to remove the extra part, and then complete the accurate reconstruction of the aorta by calculation (Figure 2A). The “FitCenterline” function was used to fit the centerline of the reconstructed model with a smoothing factor of 0.5 (Figure 2B). Subsequently, the reconstructed image data were exported from Mimics to Geomagic wrap 2021 for smoothing and surfacing (Figure 2C), of which the STL files were converted to IGES format and sutured in Ug12.0 software (Figure 2D). Finally, the desired cross-sections were cut out perpendicular to the centerline at the ascending aorta, proximal arch, distal arch, isthmus, and descending aorta (Figure 3), and a total of 465 cross-sectional images were acquired for the 93 abovementioned samples.

### 2.5. Intelligent image processing technology

Using python (version 3.7) as the programming language, we mainly apply two intelligent image processing techniques, image segmentation and contour detection, to extract the region of interest (ROI) of cross-sectional images and the pixel point coordinates of contours.

Color segmentation or threshold segmentation, semantic segmentation and edge detection are three commonly used image segmentation methods. In this paper, we implement color segmentation using OpenCV to extract the ROI of cross-sectional images. BGR color space is the default color space used by OpenCV to read color images, which mixes chroma and luminance and has poor uniformity. However, HSV color space only uses channel “H” to describe colors, which ensures color uniqueness and is more suitable for specifying color segmentation than BGR color space. Therefore, the first step is to apply the OpenCV's cv2.cvtColor(input_image, flag) function to convert the BGR color space to HSV color space, with the flag parameter set to cv2.COLOR_BGR2HSV (Equation 1).

(1) $$\begin{array}{rl} h= & \{\begin{array}{cc} 0^{\circ },\; & if\;max=min\;\\ 60^{\circ }\times \frac{g-b}{max-min}+0^{\circ },\; & if\;max=r\;and\;g\geq b\;\\ 60^{\circ }\times \frac{g-b}{max-min}+360^{\circ }, & if\;max=r\;and\;g<b\;\\ 60^{\circ }\times \frac{b-r}{max-min}+120^{\circ }, & if\;max=g\;\\ 60^{\circ }\times \frac{r-g}{max-min}+240^{\circ }, & if\;max=b\; \end{array}\\ s=\; & \{\begin{array}{cc} 0,\; & if\;max=0\\ \frac{max-min}{max}=1-\frac{min}{max}, & otherwise\; \end{array}\;\\ v=\; & max \end{array}\;$$

**Figure 4:**
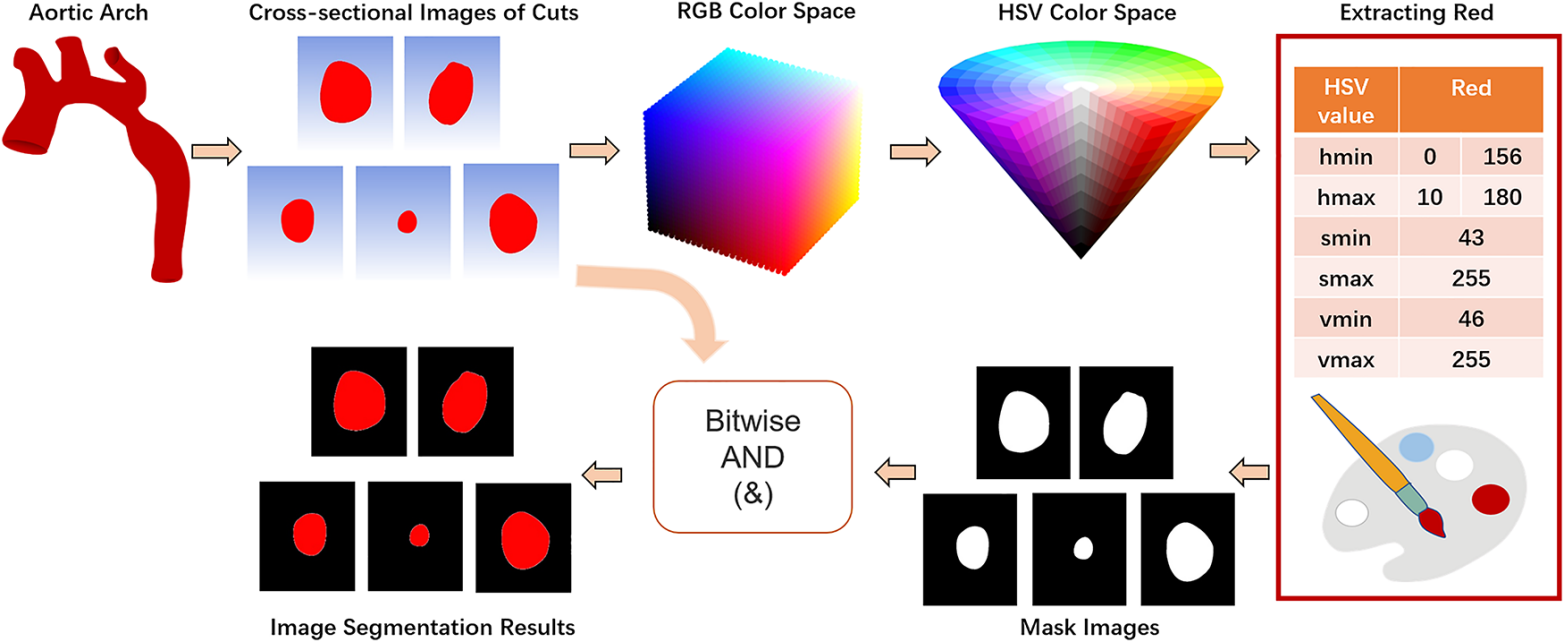
Color segmentation process.

Let (*r*, *g*, *b*) be the red, green, and blue coordinates of a color, respectively, whose values are real numbers between 0 and 1, where max is equal to the largest of *r*, *g*, *b*, and min is equivalent to the smallest of *r*, *g*, *b*. Subsequently, according to the HSV component model, the range of red color is set from [0,43,46] to [10,255,255] and [156,43,46] to [180,255,255]. Based on the two thresholds of red, the mask is constructed separately using the cv2.inRange() method, and after stitching the mask interval, it is eroded and dilated as well as a Gaussian filter with 3 × 3 kernel added. Finally, the original image and the mask are subjected to bitwise summing operations to realize the segmentation of the image (Figure 4).

Extracting the pixel point coordinates of the target image contours is essential to determine the cross-sectional center of mass. The cv2.findcontours() function in OpenCV is a simple and efficient method commonly used for contour detection, but since it accepts binary images as parameters, the read image needs to be converted into a grayscale map first, and then the image is binarized by the cv2.threshhold() function. Set the third parameter of the function, “Approximation of contours”, to cv2.CHAIN_APPROX_NONE to obtain the coordinates of all boundary points of the contour. Then, the function cv2.moments() is used to find the image moments (Equation 2), and the geometric center of the target region is determined by the first-order moments (Equation 3).

(2) $$M_{\;pq}=\overset{\infty }{\underset{-\infty }{\int }}\overset{\infty }{\underset{-\infty }{\int }}x^{p}y^{q}f(x,y)dxdy\;$$

(3) $$\{\bar{x},\bar{y}\}=\left\{\frac{M_{10}}{M_{00}},\frac{M_{01}}{M_{00}}\right\}$$

**Figure 5:**
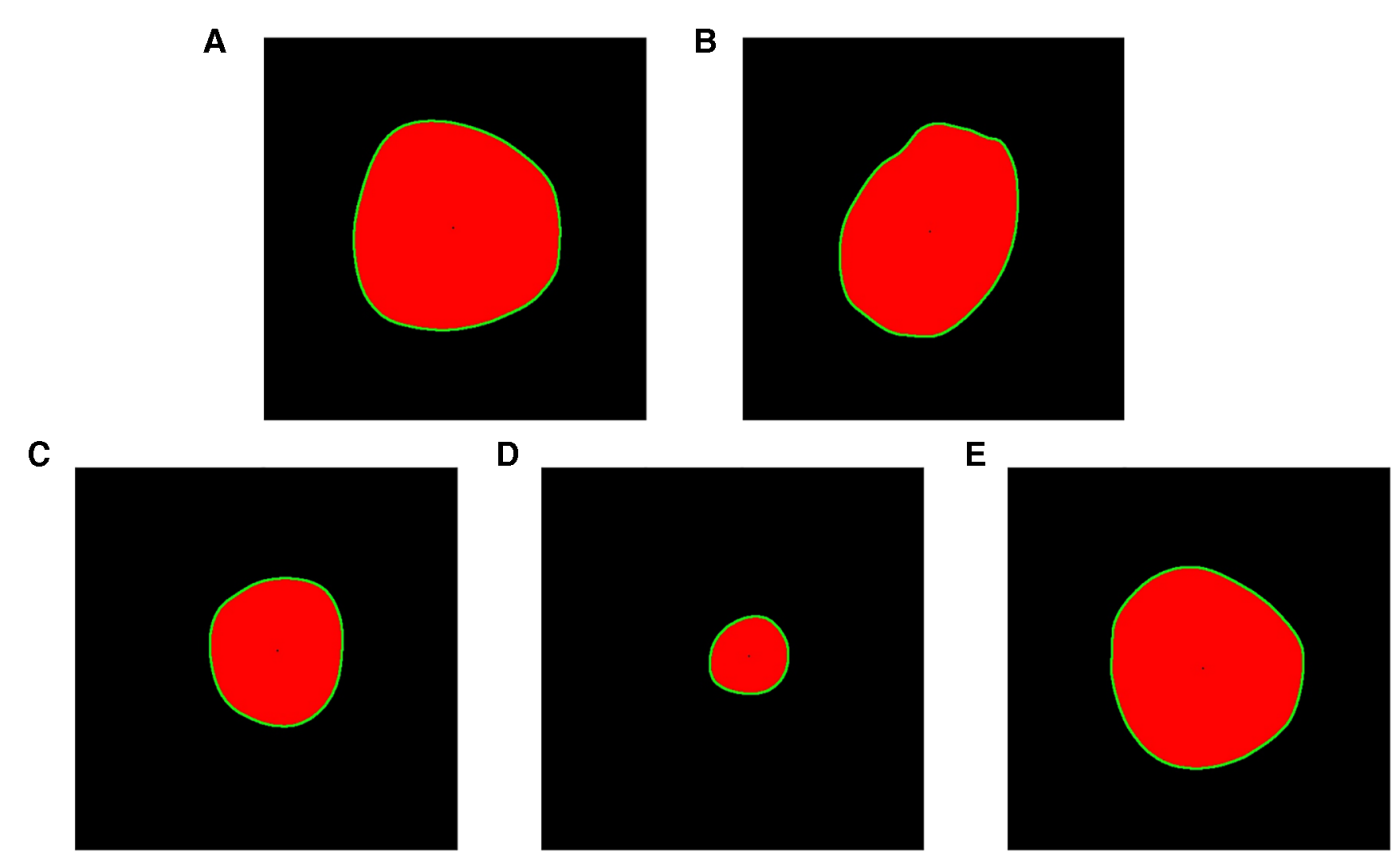
Drawing contours and geometric centers. (**A**) Ascending aortic cross section. (**B**) Proximal transverse arch cross section. (**C**) Distal transverse arch cross section. (**D**) Aortic isthmus cross section. (**E**) Descending aorta cross section.

Where *p*, *q* = 0, 1, 2 …, *f* (*x*, *y*) represents a two-dimensional image, and (*x*, *y*) is the spatial coordinate. Lastly, using cv2.drawcontours() and cv2.circle() to visualize the contours and centroids (Figure 5).

### 2.6. Intelligent measurement

Obtaining the pixel pitch information of an image is an important basis for measuring medical images. In this section, the minimum diameter of the cross section is measured in the following four steps.

(1) Dividing the contour point quadrant: Translate the image pixel coordinate system so that the center point is the origin, and divide the quadrant for the contour point according to the positive and negative signs of the horizontal and vertical coordinates.
(2) Coordinate transformation: First, the pixel point coordinates are represented in complex form, and then they are transformed into polar coordinates (*ρ*, *θ*) with the polar() method of the cmath module.
(3) Find the diameter: The diameter must pass through the geometric center, and the points connected on the same coordinate axis can be defined as the diameter, or the points with equal slopes in quadrants one and three and two and four can be connected as the diameter. The calculation and comparison of slopes are realized by tan() and isclose() methods of the math module, respectively. Note that the pixel points are small and the number of diameters found by relying only on the slope being exactly equal is unideal, so the abs_tol parameter of the isclose() function is set to 0.0256 to ensure that the slopes are equal within a certain tolerance to build more diameters.
(4) Calculate the minimum diameter: the polar diameters (*ρ*) of points with equal slopes are summed to obtain the length of the cross-sectional diameters, and the minimum diameter (in pixels) can be found by the min() function. The length of a 10 mm long scale image is 379 pixels, and the calculation yields an image pixel spacing of approximately 0.026 mm, which leads to the physical length of the smallest diameter (in mm).

### 2.7. Statistical analysis

All statistical analyses were performed using IBM SPSS Statistics, version 26 (IBM Corp). Data were analyzed as continuous variables throughout the study, which were displayed as the median (first quartile, third quartile) unless otherwise specified. Shapiro-Wilk tests were used to assess data distribution and normality. When the data had a normal distribution, two measurements comparisons were conducted using paired t-tests; Otherwise, Wilcoxon signed-rank tests were used. *P*-values were less than 0.05 (two-sided) were considered indicative of statistical significance. The consistency of intelligent diagnostic results with the gold standard was tested by the kappa test (kappa ≥ 0.75 for good agreement, 0.75 < kappa < 0.4 for fair agreement, and kappa < 0.4 for poor agreement).

## 3. Results

### 3.1. The difference between manual and intelligent measurement data

**Figure 6:**
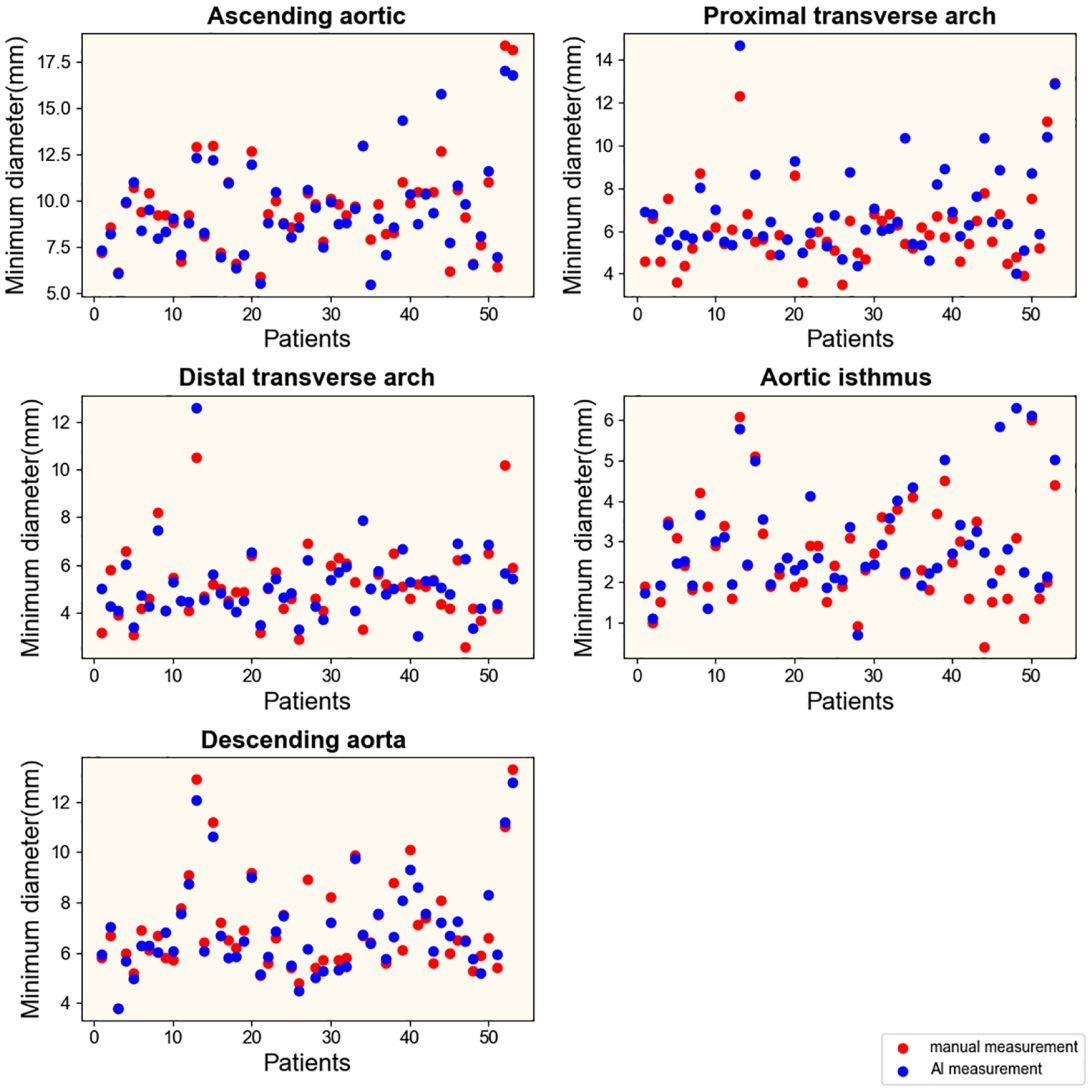
Results of measurements in patients with CoA. Manual and intelligent measurements were used to measure the minimum aortic diameter of 53 patients with CoA.

**Figure 7:**
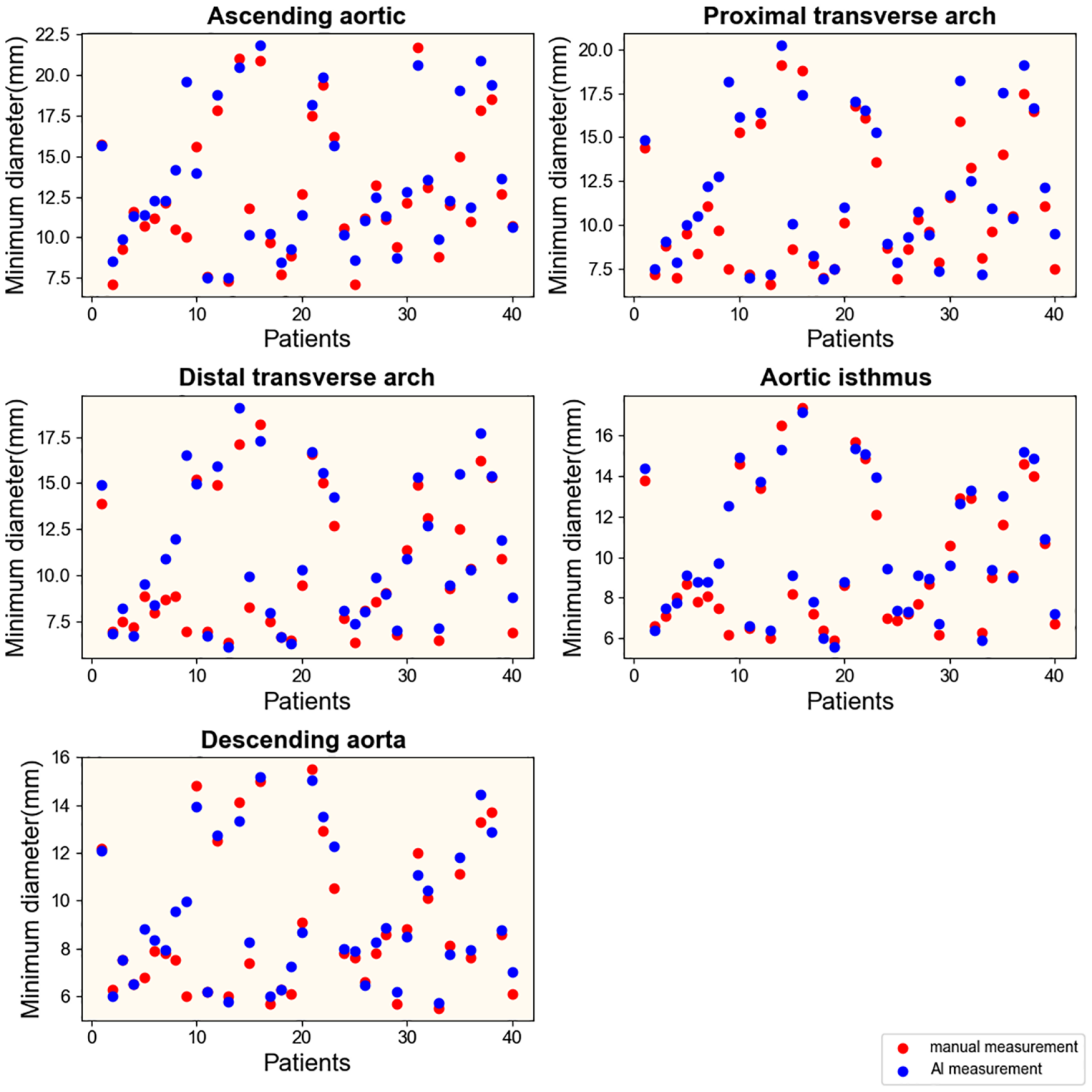
Results of measurements in patients without CoA. Manual and intelligent measurements were used to measure the minimum aortic diameter of 40 patients without CoA.

To explore the rapid and intelligent methods for the diagnosis of coarctation of the aorta, the minimum diameter of the ascending aorta, the proximal arch, the distal arch, the isthmus, and the descending aorta were measured, respectively, by intelligent image processing technology and traditional manual measurement method. Figures 6, 7 show the scatter data of the minimum diameter of the aorta measured by these two methods in 53 patients with CoA and 40 patients without CoA, respectively. By comparing the measured results of the corresponding parts of the patients (Figures 6, 7), although the distribution of a few measured values was similar, there were some differences in most of them, with the maximum difference being about 10 mm. Subsequently, the Wilcoxon signed-rank test was further used to compare the differences in the distribution of measurements (Table 1). In patients with CoA, the minimum diameter of the distal arch measured by the two methods was the closest (*p* = 0.968), and there was no significant difference in the measurement results at the descending aorta (*p* = 0.158). Whether people have CoA or not, both methods showed significant differences in the measurement results of the remaining parts (all *p* < 0.05).

**Table 1:** Measurement results of two measurement methods.

Characteristics	Manual measurement	AI measurement	Difference	*p* value
CoA_AOA, median, IQR (mm)	9.3 (8.0, 10.5)	8.8 (7.9, 10.5)	0.12 (−0.24, 0.72)	0.040
CoA_D1, median, IQR (mm)	5.7 (5.1, 6.7)	6.1 (5.6, 7.8)	−0.65 (−1.45, 0.12)	<0.001
CoA_D2, median, IQR (mm)	5.0 (4.2, 5.9)	5.0 (4.3, 5.7)	−0.03 (−0.40, 0.42)	0.968
CoA_D3, median, IQR (mm)	2.4 (1.9, 3.4)	2.5 (2.1, 3.5)	−0.14 (−0.40, 0.20)	0.026
CoA_DA, median, IQR (mm)	6.5 (5.7, 7.7)	6.4 (5.8, 7.5)	0.15 (−0.22, 0.49)	0.158
No_AOA, median, IQR (mm)	11.7 (9.8, 15.7)	12.2 (10.2, 17.5)	−0.55 (−1.30, −0.13)	0.019
No_D1, median, IQR (mm)	9.7 (7.8, 14.3)	10.9 (8.4, 16.3)	−0.46 (−1.24, 0.08)	<0.001
No_D2, median, IQR (mm)	8.9 (7.1, 13.7)	10.1 (8.0, 15.2)	−0.38 (−0.65, 0.20)	<0.001
No_D3, median, IQR (mm)	8.4 (6.9, 12.9)	9.1 (7.4, 13.6)	−0.21 (−0.58, 0.22)	0.001
No_DA, median, IQR (mm)	7.8 (6.4, 11.8)	8.4 (7.1, 12.0)	−0.46 (−0.91, 0.23)	0.034

IQR, Interquartile Range; CoA_AOA, CoA_D1, CoA_D2, CoA_D3, and CoA_DA are the diameters of the ascending aorta, proximal arch, distal arch, isthmus, and descending aorta in patients with CoA, respectively; No_AOA, No_D1, No _D2, No _D3, and No _DA are the diameters of the ascending aorta, proximal arch, distal arch, isthmus, and descending aorta in patients without CoA, respectively;.

Generally, using intelligent image processing technology to automatically construct the minimum diameter and obtain the measured value in pixels is more conducive to capturing the detailed features of the image than manually. It is more objective and authentic, and measurement results may be more accurate. However, the analysis of the accuracy of the measurement method mainly depends on the final diagnostic results.

### 3.2. Diagnostic results based on two measurement methods

**Figure 8:**
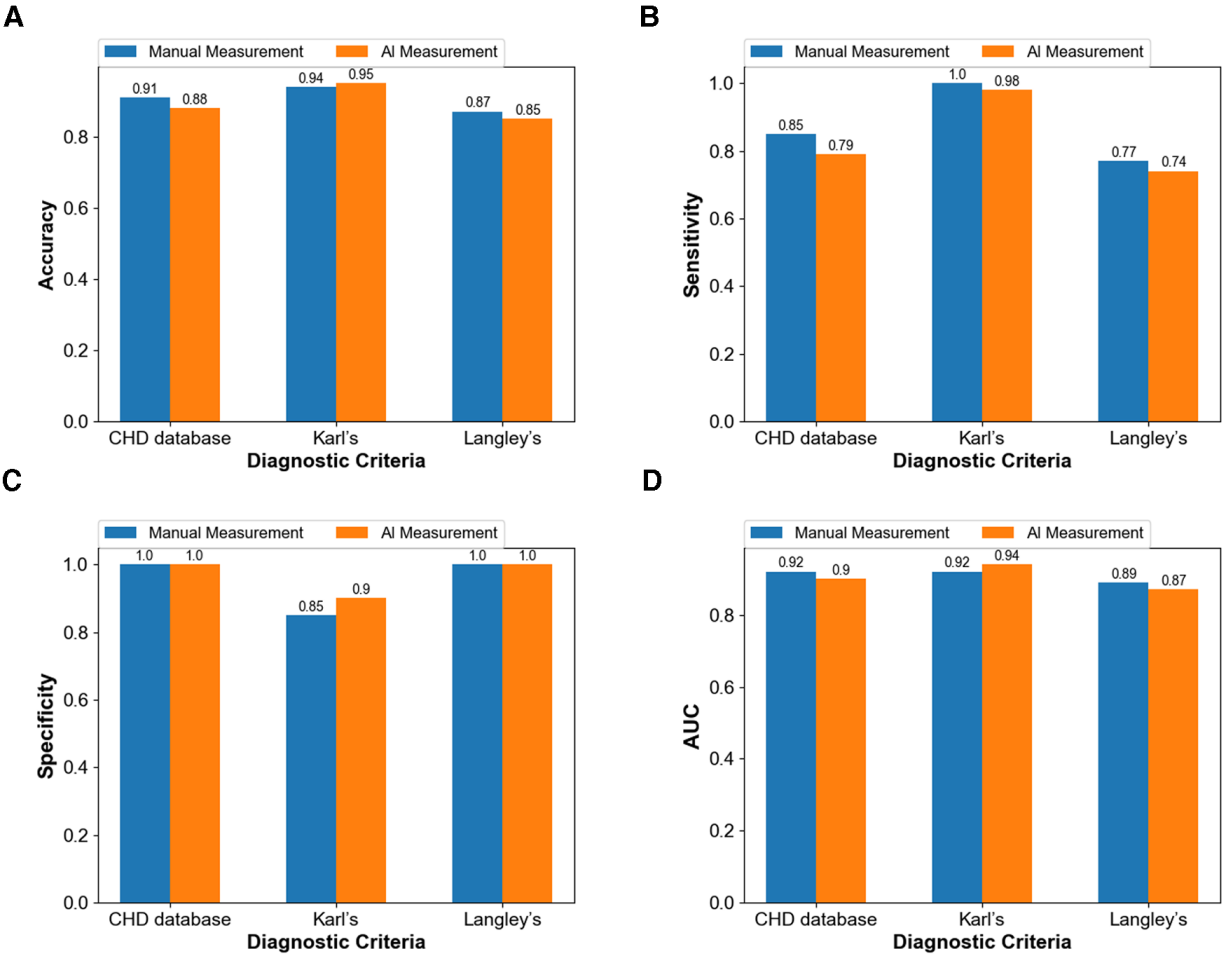
Comparison between the diagnostic capacity of the manual method and of the intelligent measurement (**A**) diagnostic accuracy (**B**) diagnostic sensitivity (**C**) diagnostic specificity (**D**) diagnostic AUC.

The ultimate purpose of the measurement based on the two methods is still to make a reliable diagnosis. Therefore, using the diagnostic results of surgery as the gold standard in this study, four indexes including accuracy, sensitivity, specificity, and AUC (area under the curve) were used to evaluate the accuracy of the traditional and intelligent methods in the diagnosis of aortic coarctation. The measured values obtained using the two methods were respectively applied to the three diagnostic criteria, and the final diagnostic accuracy, specificity, and AUC values were all higher than 85% (Figures 8A,C,D). The intelligent measurement showed better performance in the above three indicators when using Karl's classification. However, in the other two standards, the performance of the traditional measurement method is better than that of the intelligent. Manual measurements always showed higher diagnostic sensitivity (Figure 8B) than intelligent measurements, with both methods achieving up to 100% specificity (Figure 8C) in diagnoses based on the CHD database classification and Langley's classification.

Even in terms of measurements are different, the intelligent image processing technology combining image segmentation and contour detection shows similar diagnostic results to traditional, and even more accurate than traditional methods in some aspects. Moreover, the diagnostic results of the method applied to the three criteria agreed well with the gold standard (all kappa ≥ 0.8). This result proves that the computer-intelligent measurement method can be successfully applied in the diagnosis of CoA according to specific standards.

### 3.3. Comparative analysis of diagnostic criteria

**Figure 9:**
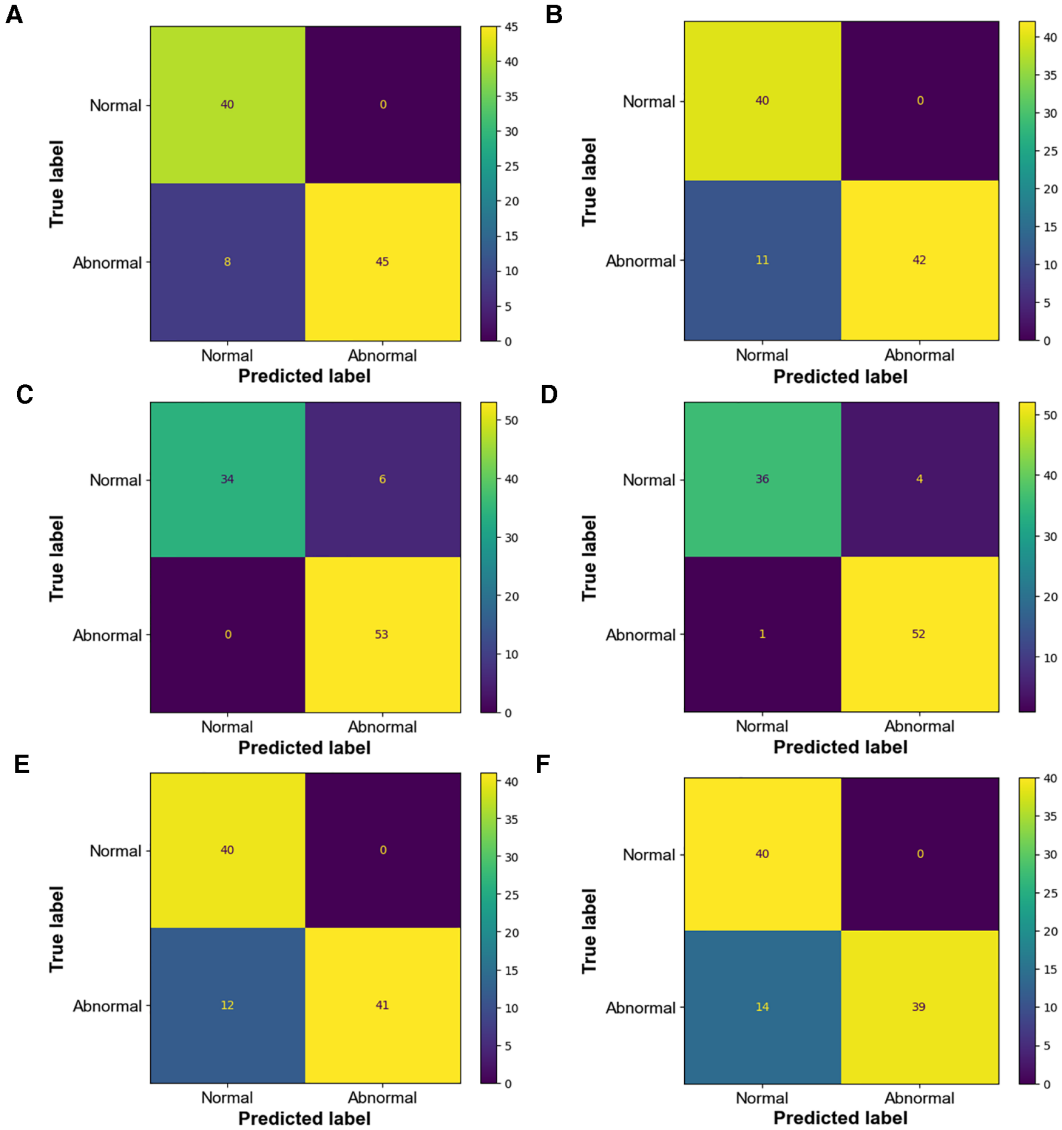
Confusion matrix of diagnostic results (**A**) confusion matrix of manual measurement diagnosis based on CHD database classification (**B**) confusion matrix of intelligent measurement diagnosis based on CHD database classification; kappa = 0.84 (**C**) confusion matrix of manual measurement diagnosis based on Karl's classification (**D**) confusion matrix of intelligent measurement diagnosis based on Karl's classification; kappa = 0.93 (**E**) confusion matrix of manual measurement diagnosis based on Langley's classification (**F**) confusion matrix of intelligent measurement diagnosis based on Langley's classification; kappa = 0.8.

Because there is no uniform standard for the diagnosis of CoA at present, the selection of different diagnostic criteria will lead to inconsistent diagnostic results, which will have a direct impact on the intervention and treatment of patients. Therefore, this part compares and analyzes the diagnostic criteria by visualizing the confusion matrix (Figure 9) of the diagnostic results and showing the diagnostic efficiency corresponding to the diagnostic criteria.

In this research, the diagnosis of coarctation of the aorta was made using the geometric-based imaging diagnostic criteria specified in the “Consensus of Experts in the Surgical Treatment of Congenital Heart Disease in China”. However, there are still some controversies concerning these diagnostic criteria in clinical practice. First, the CHD database classification emphasized morphological changes at multiple sites, but most children met only part of the conditions. We identified those who met either of conditions as patients with CoA. Secondly, since Karl's classification is often used for newborns and small infants, in order not to be restricted by this age stage, this standard was optimized according to the weights of patients (kg) in our study to improve the universality:

0 < weights < 6: weights + 1
6 ≤ weights < 10: weights
20 ≤ weights < 30: weights/2
30 ≤ weights < 40: weights/2.5
weights ≥ 40: weights/3

Langley's classification has high rates of misdiagnosis and low diagnostic accuracy (Tables 2, 3) based on both manual and intelligent measurement methods. The accuracy and sensitivity of Karl's classification were the highest among the three diagnostic criteria (Tables 2, 3). The sensitivity reached 100% in the diagnosis based on manual measurement methods (Table 2). The specificity of the CHD database classification and Langley's classification were as high as 100% (Tables 2, 3). When using traditional methods to measure, the AUC of the CHD database classification and Karl's classification were equal (Table 2), but when using intelligent methods, Karl's classification achieved a higher AUC (94%) (Table 3). In general, Karl's classification has the best diagnostic effect.

**Table 2:** Comparison of diagnostic criteria based on manual measurement.

Evaluation indicators	CHD database classification	Karl's classification	Langley's classification
Number of correct diagnoses	85	87	81
Number of misdiagnoses	8	6	12
Total number	93	93	93
Accuracy	0.91	0.94	0.87
Sensitivity	0.85	1.00	0.77
Specificity	1.00	0.85	1.00
AUC	0.92	0.92	0.89

Areas under receiver operating characteristics curve (AUC).

**Table 3:** Comparison of diagnostic criteria based on intelligent measurement.

Evaluation indicators	CHD database classification	Karl's classification	Langley's classification
Number of correct diagnoses	82	88	79
Number of misdiagnoses	11	5	14
Total number	93	93	93
Accuracy	0.88	0.95	0.85
Sensitivity	0.79	0.98	0.74
Specificity	1.00	0.90	1.00
AUC	0.90	0.94	0.87

Areas under receiver operating characteristics curve (AUC).

Although the CHD database classification and Karl's classification have been changed and optimized, and good diagnostic results have been achieved, it is still a preliminary exploration and need to be gradually evidence-based in practice.

## 4. Discussion

The failure of early diagnosis of aortic coarctation of the aorta leads to high morbidity and mortality (22). In this study, an intelligent method for aortic arch measurement is provided by combining clinical medicine, computer three-dimensional image reconstruction, and intelligent image processing technology. It has been applied to the three clear imaging diagnostic criteria, has achieved a high level of diagnostic efficiency, and is superior to traditional diagnosis in some aspects. Furthermore, by comparing and analyzing the three diagnostic criteria, it was found that Kral's classification showed high sensitivity and specificity in both methods.

### 4.1. Automatic construction and measurement of aortic diameter

Image processing technology is playing an increasingly important role in the diagnosis of cardiovascular diseases (23, 24). The application of contour detection and other image processing techniques to quickly find the geometric center of aortic slices, automatically construct the diameter, and output the measured value of the minimum diameter is a great advantage of this study. Although the final manual measurements are different from the measurements obtained by this method, it has little effect on the diagnosis of CoA. Compared with manual diagnosis, first, the use of this intelligent measurement method optimizes the doctor's diagnosis time and realizes accurate and rapid diagnosis of CoA. Second, this paper measures the length of the minimum diameter of the aorta in pixels (the basic unit of digital images), which can be extended to other related medical image measurements, providing the possibility of obtaining a more accurate medical examination and test data. Finally, the intelligent method can provide more objective and realistic aortic measurement results, which are not affected by personal experience and reduce subjectivity.

### 4.2. Diameter measurements were combined with CoA diagnostic criteria

It is another feature of this study to use the diameter values obtained by manual and intelligent methods for CoA imaging diagnosis. Although the “Consensus of Experts in the Surgical Treatment of Congenital Heart Disease in China” has defined four diagnostic criteria, the diagnostic criteria for CoA are still not uniform. Early studies rarely combined this value with relevant standards for disease diagnosis after obtaining diameter measurements. In this current study, we compared the diagnostic results obtained by the two measurement methods with the diagnostic criteria. Either way, Karl's classification has better diagnostic performance than the other criteria. Based on this result, a practical recommendation can be made for the clinician: to determine whether a patient has CoA after CTA examination, Karl's classification should be preferred, and if necessary, use the CHD database classification to support the diagnosis.

### 4.3. Development prospect and limitation

Although the peri-operative mortality of CoA has been decreased to less than 3% (25), the incidence of its postoperative complications is still at 36%∼68.8% (26). Therefore, regular prognostic follow-up and prediction of the risk of adverse events in patients with CoA are vital. Huijun Xiao retrospectively analyzed data related to 27 infants with isolated CoA who underwent surgical correction and identified predictive variables associated with surgical outcomes (27). Yan Gu et al. (28) used daily clinical practice data from 514 patients with CoA to develop a model for predicting adverse events at 30 days postoperatively or during hospitalization adverse events, with a significant improvement, compared with two commonly used risk assessment strategies (the ABC score and RACHS-1). If the rapid and intelligent diagnostic protocol mentioned in this study can be combined with this prognostic risk prediction strategy, it will have a beneficial impact on improving the cure rate of CoA and reducing the risk of prognostic complications and death.

One limitation of this study is that the relevant sections were obtained perpendicular to its centerline after reconstructing the aorta using a manual cutting method, which may produce some errors. This may be why there is a discrepancy between intelligent and manually measured aortic data. We will try to use artificial intelligence to achieve automatic sections in the follow-up study to reduce manual intervention.

## 5. Conclusion

The current study used 3D reconstruction and intelligent image processing technology combined with CT examination imaging diagnostic criteria to diagnose CoA, and the diagnostic effect was the same as that of traditional manual measurements, which not only alleviated the problem of the insufficient number of senior clinicians but also improved the diagnostic speed and reduced the subjectivity of aortic measurements. The proposed intelligent measurement method is a promising technology, which is expected to be extended to the quantitative measurement of other medical images and improve the efficiency of clinical decision-making.

## Data Availability

The raw data supporting the conclusions of this article will be made available by the authors, without undue reservation.
